# Supplementary material for: Geometric Constraints Dominate the Antigenic Evolution of Influenza H3N2 Hemagglutinin
Source: PLoS Pathog. 2015 May 28;11(5):e1004940. doi: 10.1371/journal.ppat.1004940 (PMC4447415; doi:10.1371/journal.ppat.1004940)
Supplement: S1 Text — (PDF) [file ppat.1004940.s002.pdf]

Supporting Text: Geometric constraints dominate the antigenic evolution of influenza H3N2 hemagglutinin

Austin G. Meyer and Claus O. Wilke

Mapping Immune Epitope Database (IEDB) data

We asked whether there is experimental evidence that the human immune system displays a bias towards particular regions of hemagglutinin; such a bias would traditionally be called an immune epitope. We obtained all available human B cell epitopes for H3 hemagglutinin from the Influenza Research Database (IRD). There were two broad categories of epitope data available in the epitope database. One was non-linear epitopes and the other was linear epitopes. In addition, the IRD splits epitopes into B and T cell epitopes and also into the various species whose immune system was tested. Here, we were interested in antibody-driven immune escape, and hence we focused on B cell epitopes.

We initially considered all available B cell epitope data in our analysis, inluding human and non-human, because non-human data have traditionally been part of the data considered for epitope definition in influenza (see e.g. Ref. [10] in the main text). In the database, there were two types of B cell epitopes available for hemagglutinin: linear and non-linear. For humans, there were 31 separate epitope entries consisting of 26 non-linear and 5 linear. For B cell epitopes, every linear human epitope was also listed in the IRD as the linear epitope of some other species, most commonly mouse but also, for example, ferret. For non-humans, there were 134 available epitope entries consisting of 47 non-linear and 87 linear. The non-linear epitopes were provided as site numbers, so that mapping them onto the protein structure was trivial.

For linear epitopes, we started with the short sequence fragments; each of the fragments was between 5 and 40 amino acids in length. We tried initially to map the fragments onto the emblematic A/Aichi/2/1968 sequence, but it turned out the epitope fragments were actually generated from disparate strains along the H3N2 lineage. The differences between the original founder strain and the fragments meant that we could not accurately map the vast majority of short peptides onto a single sequence. Instead, we took the entire curated and pre-aligned set of 3854 sequences that we used in the evolutionary rate calculations. We then aligned the fragments with MAFFT using a very strong opening penalty of 10. We visually checked to be sure all of the 87 fragments aligned reasonably well to the full H3 alignment. Then, as with the non-linear epitopes, we counted the number of times each site was hit and the co-occurrences of sites hit by the same antibody. Since the 5 linear human epitopes were a subset of the 87 non-human linear epitopes, we dropped the linear human epitopes from further analysis.

Comparing historical epitope groups to IEDB immune epitope data

For both linear and non-linear epitope data, we counted for each site in hemagglutinin how often it appeared in each epitope data set. We then compared these epitope counts to the historical epitope sites (Bush 1999, Ref. [4] in the main text). We first considered the human, non-linear epitope data. We found some overlap between the historical sites and the IEDB non-linear epitope counts (Fig. S3). Each of the four largest peaks (i.e., the four regions with strongest IEDB evidence of belonging to epitopes) was at least partially captured by the historical epitope

groups. The majority of historical epitopes A and B were represented in the non-linear epitope data; epitope A had 16 of 19 sites appear in the IEDB data and epitope B had 13 of 22. By contrast, many of the historical sites had no support among the available non-linear epitope data. Of 131 historical epitope sites, only 52 appeared at least once in the IEDB data set. Historical epitopes C, D, and E were particularly poorly represented as a fraction of their size, with only 8 of 27, 13 of 41, and 2 of 22 sites having support, respectively. Among the discrepancies between historical and IEDB immune epitopes, an important deviation came from the HA2 chain. By historical convention, no sites from HA2 were included among the hemagglutinin epitopes; as a result, there were a large number of sites appearing in the IEDB set that were not defined as historical epitopes.

To understand to what extent the IEDB non-linear epitope sites separated into distinct groups, we performed a clustering analysis (Fig. S4A). We considered all sites in the IEDB epitope dataset as nodes of a graph, and we drew an edge between any two sites that appeared within the same accession number of the immune epitope database (IEDB). Then, we colored the nodes according to their classification in the historical epitope groups. We found that the non-linear IEDB epitope data was able to partially reconstruct the historical epitope groups. In particular, historical epitopes A, B, and D clustered well, with only two sites from A and one site from D being completely disconnected from the rest of the epitope. By contrast, epitopes C and E did not at all recapitulate any IEDB data. In addition, as previously shown in Fig. S3, there was a relatively large number of IEDB epitope sites that were not accounted for by the historical epitope definition (Fig. S4A).

In addition to antibody connectivity (accession number clustering), given the structural nature of antibody neutralization, we expected that any correct grouping of epitope sites would display some ability to cluster in three dimensional space. Thus, we calculated the distance from each C_α (the α-Carbon atom in the polypeptide backbone) site in hemagglutinin to every other C_α. We then constructed a graph where we connected any two nodes in the non-linear IEDB epitope data set with an edge if the two corresponding sites were less than 10 Å apart in the 3D structure (Fig. S4B). Again, we colored the nodes by their historical groupings. For these spatial clusters, the historical epitopes were poorly grouped within the non-linear epitope data. All historical epitopes fell into at least two disjoint sets separated by sites not belonging to the same epitope, and no single visible spatial cluster corresponded to a single historical epitope. We concluded that the historical epitope definitions largely failed to spatially cluster within the sites for which we had IEDB immune epitope data.

We next considered the non-human, non-linear epitopes. We found that there was no clear connection between the non-human non-linear epitope data and historical epitopes groups (Fig. S5); in addition, several of the accessions listed individual sites, which thus did not provide any information about epitope groupings. We also considered IEDB non-human linear epitopes. By contrast to the non-linear epitopes, the linear epitope set covered nearly every site in the entire hemagglutinin protein (Fig. S6). However, the most represented sites in the IEDB epitope dataset (near site 100) were almost completely missed by the historical epitope sites. Furthermore, there was a substantial portion of IEDB sites near the N-terminal region of HA1 that was completely absent from the historical sites. When clustering the data by shared accession numbers, we found that the IEDB linear epitope data did not at all resemble historical epitope groups (Fig S7). Moreover, since the IEDB linear epitopes covered almost every site in HA, it

was not clear that they represented any particular immune-system bias. In fact, we expect that the experimental practice of expressing short linear peptides and testing them against antibody binding will generally produce many false positives, sites that are included in the peptide but not actually bound by an antibody.

Because neither the linear nor the non-linear non-human epitope sites appeared particularly informative, and because in general it is not clear that non-human immune data are relevant to human epitope grouping, we disregarded all non-human epitope data for the remainder of this study. We thus assumed that the non-linear human B cell epitopes represent the true immune epitope sites. We acknowledge that at least some of the sites included among the linear epitopes should likely be included among the true immune epitopes. However, these sites would first have to be verified by non-linear mapping.

Regrouping epitope sites with IEDB data

Even though the historical epitopes were able to partially reconstruct the IEDB epitope clusters, a simple visual inspection suggested more natural groups than those used in the historical set (Fig. S4A). Thus, we re-grouped the IEDB human non-linear epitopes into the most obvious possible groupings (Fig. S4C and Table S2). The non-linear epitope data clustered most naturally into four distinct immune epitope regions, which we referred to as 1–4, to distinguish them from the historical epitopes A–E. Two of the four regions (IEDB regions 2 and 3, respectively) were generally very similar to epitopes A and B in the historical definitions. One of the two remaining regrouped epitopes (IEDB region 4) was vaguely similar to the D historical epitope with many sites added. The last epitope (IEDB region 1) supported by IEDB non-linear data was virtually nonexistent in all previous epitope groups. It had a few sites that were previously classified in the C epitope, and it added a large number of sites from the HA2 chain of hemagglutinin. Finally, there were eight sites that had at least one count in the non-linear epitopes and that could not be easily clustered with the other sites (Table S2).

We performed the same spatial clustering with our newly defined epitope groups as we had previously done for the historical sites. By contrast to the historical sites, we found that our groups, which were defined simply by antibody-clustered sites, partitioned almost perfectly into spatial clusters (Fig. S4D). Of the four groups we defined, all but one (IEDB region 2) was spatially connected. In addition, IEDB region 2 had only a single spatial disconnection. Our data set may be missing a single site that would flip the relevant portion of the graph and connect the two disconnected sections. In addition, the spatial graph suggests how to resolve the ungrouped sites (drawn in black) in Fig. S4C. Of these eight sites, which grouped into one pair and two triplets in Fig. S4C, two likely belong to epitope 2, two likely belong to epitope 3, and three may belong to either epitope 2 or 3. Notably, while our redefined epitopes clustered well in the 3D structure, they looked disconnected and arbitrarily chosen when plotted along the linear chain (Fig. S9).

Finally, we mapped our epitope groups onto the 3D crystal structure (Fig. S8), using the same color scheme as used in Figure S4. As expected, there was a clear spatial distribution of sites. Moreover, groups 2–4 fell into the apical domain of HA, directly adjacent to the sialic acid-binding region. Only group 1, which clearly separated from 2–4 in the clustering analysis, was located in the stem of HA.

Table S2: Groups of IEDB non-linear epitope sites. We defined epitope groups on the basis of which sites co-occurred within the same IEDB accession number. See also Fig. S4C and S1 Dataset. Sites are numbered according to their position in the mature protein.

Epitope group	Sites
1	34, 36, 53, 54, 70, 292, 295, 305, 307, 334, 363, 364, 365, 366, 379, 380, 382, 383, 384, 386, 387, 390, 391, 393, 394, 395, 397, 398, 401, 403, 404, 405, 499
2	121, 122, 123, 124, 126, 131, 133, 135, 136, 137, 138, 140, 142, 143, 144, 145, 146
3	155, 156, 157, 158, 159, 160, 187, 188, 189, 190, 191, 192, 194, 196, 223, 256
4	114, 115, 147, 148, 149, 150, 151, 152, 153, 154, 161, 162, 169, 170, 171, 172, 173, 174, 175, 176, 204, 205, 206, 208, 209, 210, 211, 212, 235, 238, 241, 242, 243
N/A	82, 83, 222, 225, 275, 276, 278

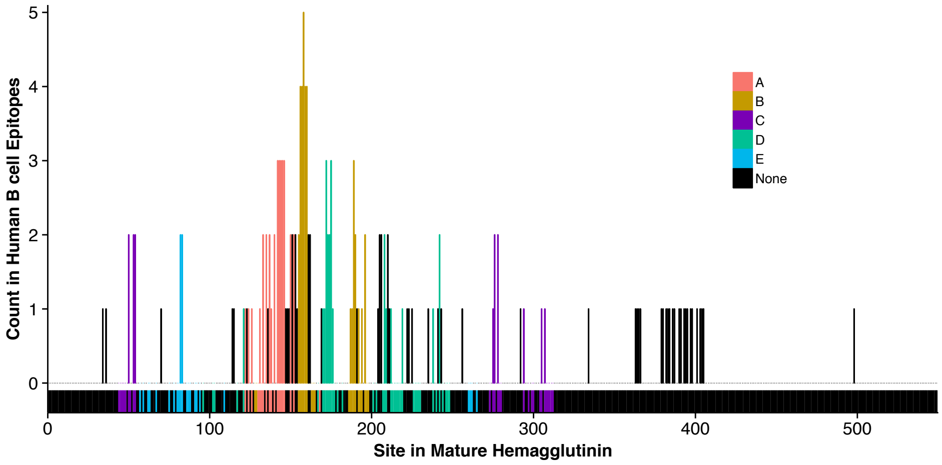

Figure S3: IEDB non-linear epitope site counts, colored by historical epitope groupings. The heights of individual bars indicate how often each site in the H3 hemagglutinin protein appears in an IEDB non-linear epitope set, and the color of each bar indicates the site's historical epitope assignment according to Bush et. al 1999 (Ref. [4] in the main text). Sites that do not appear in the IEDB set are shown with a count of zero. The rug underneath the $y = 0$ line contains all sites and visualizes the exact loation of the historical epitope sites.

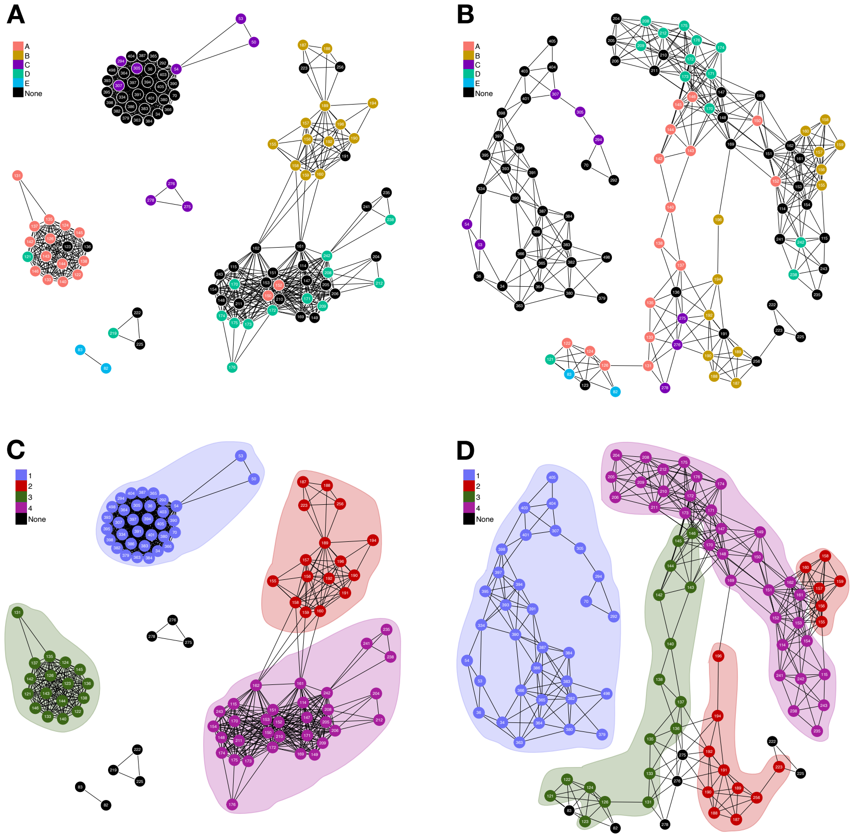

Figure S4: Clustering of IEDB non-linear epitope sites by IEDB accession number and by physical proximity in the 3D structure. (A) Non-linear epitope sites clustered by IEDB accession number and colored according to the historical epitope definition (Ref. [4] in the main text). Each node represents a site that appears at least once in the IEDB non-linear epitope data set. Two nodes are connected by an edge if they are bound by the same antibody in the Immune Epitope Database (i.e., have the same IEDB accession number). The historical epitope definitions do not correspond well to the observed clustering. (B) Non-linear epitope sites clustered by physical proximity in the 3D structure and colored according to the historical epitope definition. The same nodes as in (A) are now connected by an edge if they are within 10 Å of each other in the three dimensional structure. The historical epitope groupings do not appear clustered in 3D space. (C) Sites are clustered as in A but colored according to the most natural grouping based on antibody clustering. Colored areas are drawn to highlight the distinct clusters. (D) Site are clustered as in (B) but colored as in (C). Parts (C) and (D) show that the IEDB non-linear epitope data support the expectation that immune epitopes should group together spatially. All the sites that group by binding the same set of antibodies are also spatially connected. The only exception is the red epitope (#2), which is split into two groups. Also, there is one site that appears once in the data, but is not connected to any other sites; as a result, it could not be displayed.

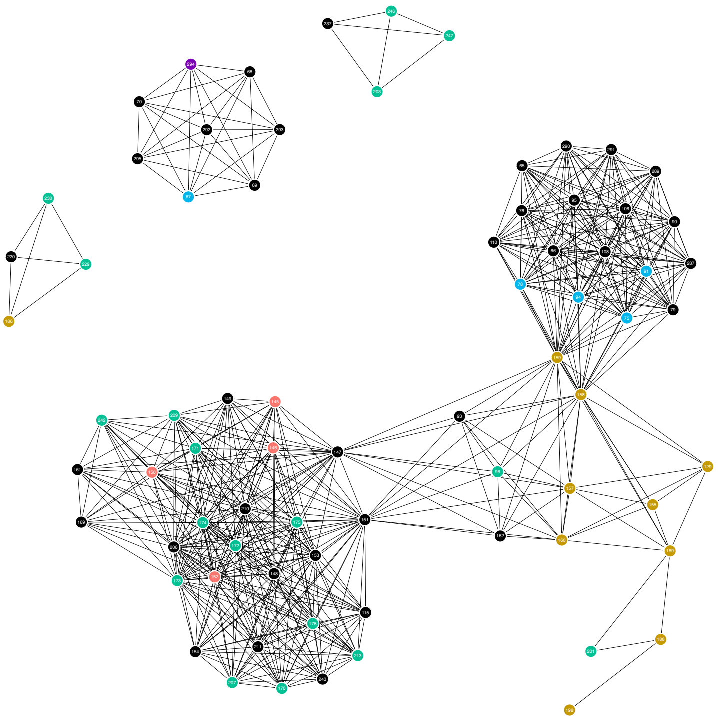

Figure S5: Clustering of IEDB non-human non-linear epitope sites by IEDB accession number. Each node represents a site that appears at least once and is connected to another site in the IEDB non-linear epitope data set. Nodes are colored according to the historical epitope definition (Ref. [4] in the main text). Two nodes are connected by an edge if they are both part of the same IEDB accession number. The historical epitope definitions do not correspond well to the observed clustering. Also, there are 13 sites that appear once in the data but are not connected to any other sites; as a result, they could not be displayed.

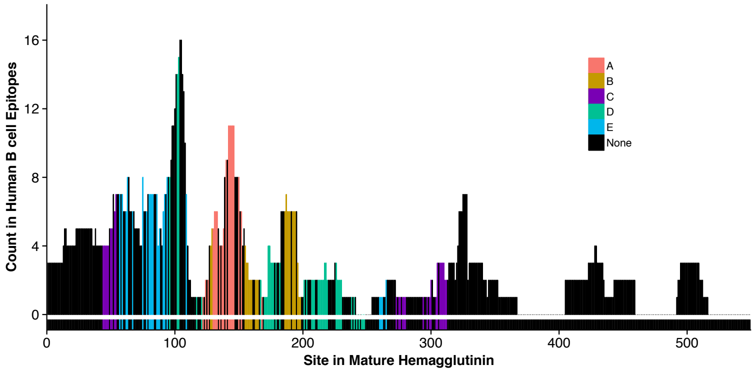

Figure S6: IEDB non-human linear epitope site counts, colored by historical epitope groupings. The heights of individual bars indicate how often each site in the H3 hemagglutinin protein appears in an IEDB linear epitope set, and the color of each bar indicates the site's historical epitope assignment according to Bush et. al 1999 (Ref. [4] in the main text). Sites that do not appear in the IEDB set are shown with a count of zero. The rug underneath the $y = 0$ line contains all sites and visualizes the exact loation of the historical epitope sites.

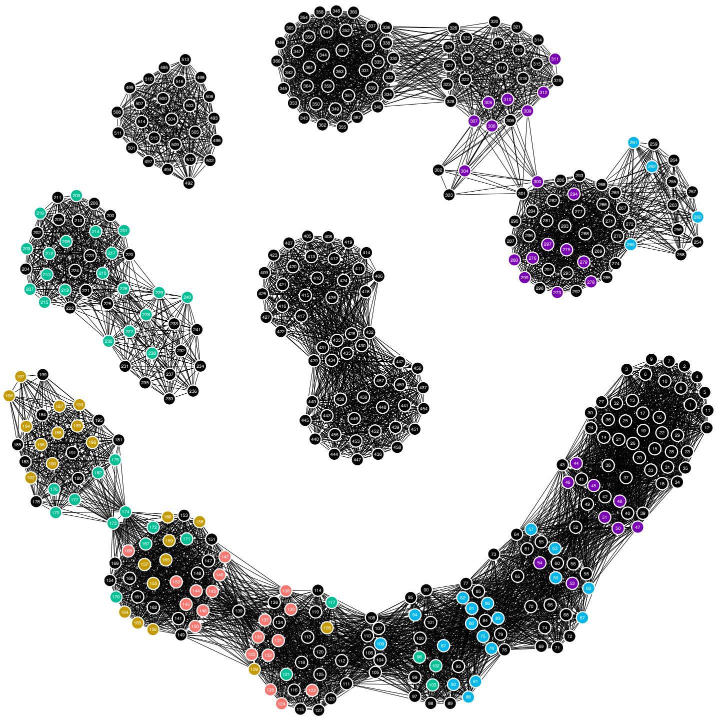

Figure S7: Clustering of IEDB non-human linear epitope sites by IEDB accession number. Each node represents a site that appears at least once and is connected to another site in the IEDB linear epitope data set. Nodes are colored according to the historical epitope definition (Ref. [4] in the main text). Two nodes are connected by an edge if they are both part of the same IEDB accession number. The historical epitope definitions do not correspond well to the observed clustering.

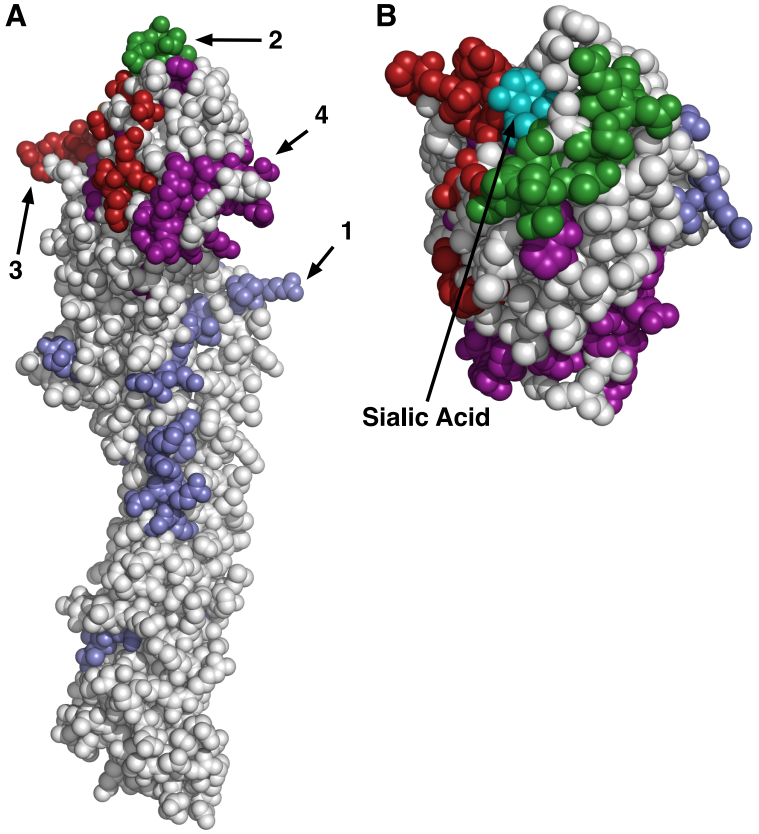

Figure S8: Location of IEDB, non-linear epitopes in the 3D structure of hemagglutinin. (A) Side perspective of hemagglutinin. Non-linear epitope sites are colored according to their group assignment, as defined in Fig. S4. (B) Top perspective of hemagglutinin, with epitope sites highlighted. The orange moiety is sialic acid: the human receptor for influenza.

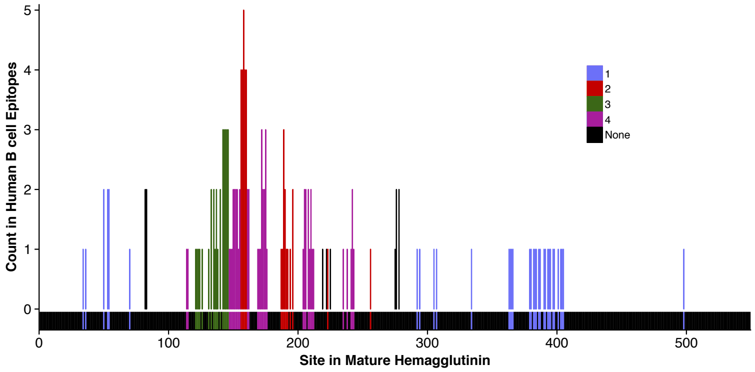

Figure S9: IEDB human non-linear epitope site counts, colored by our proposed, IEDB-based epitope groupings. The heights of individual bars indicate how often each site in the H3 hemagglutinin protein appears in an IEDB non-linear epitope set, and the color of each bar indicates the site's epitope assignment. Sites that do not appear in the IEDB set are shown with a count of zero. The IEDB-based epitopes are not clustered linearly along the sequence, but instead fall into a non-contiguous spatial arrangement.
