# Supplementary figures and images for: Geometric Constraints Dominate the Antigenic Evolution of Influenza H3N2 Hemagglutinin
Source: PLoS Pathog. 2015 May 28;11(5):e1004940. doi: 10.1371/journal.ppat.1004940 (PMC4447415; doi:10.1371/journal.ppat.1004940)

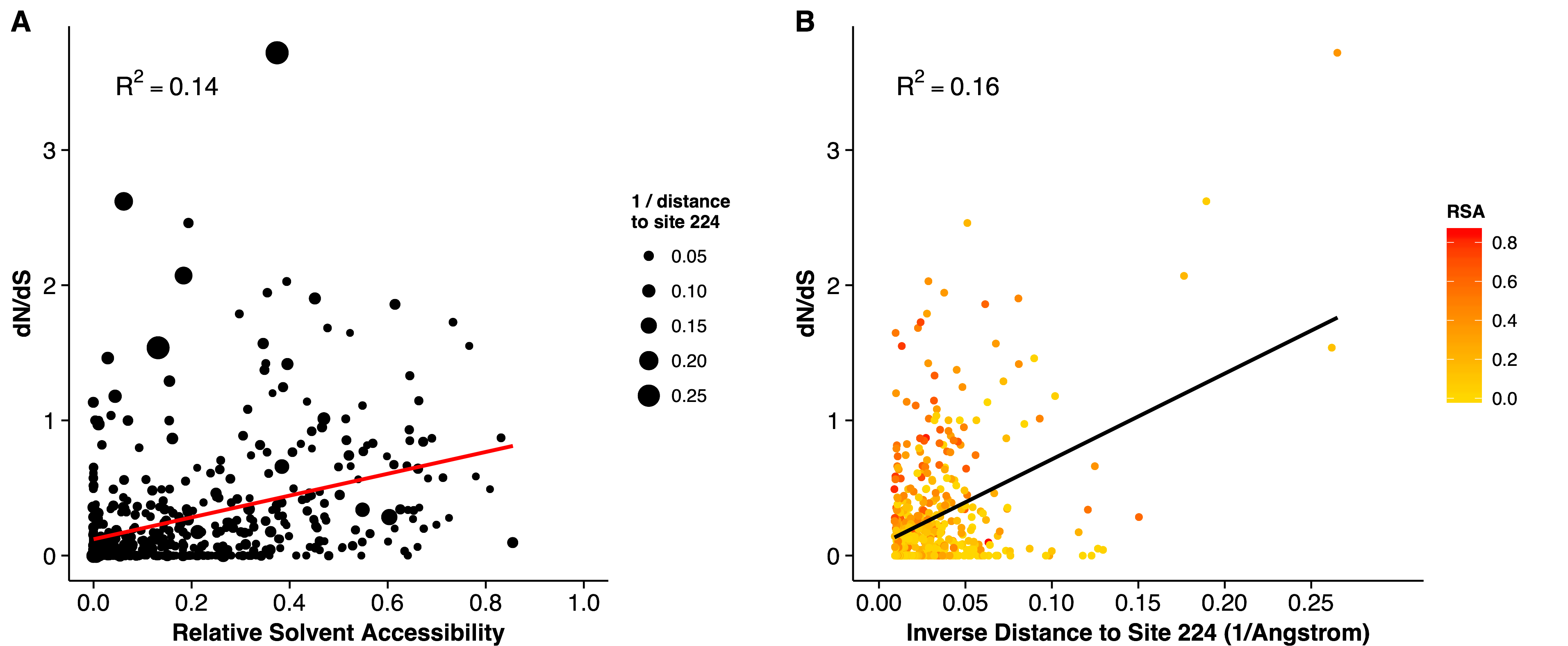

Supplement: S1 Fig — (A) dN/dS vs. RSA. The size of the dots represents 1/Distance. (B) dN/dS vs. 1/Distance. The coloring of the dots represents RSA. The distance to the sialic acid-binding region is the single strongest quantitative predictor of evolutionary rate ratio in hemagglutinin. (TIFF) [file ppat.1004940.s003.tiff]

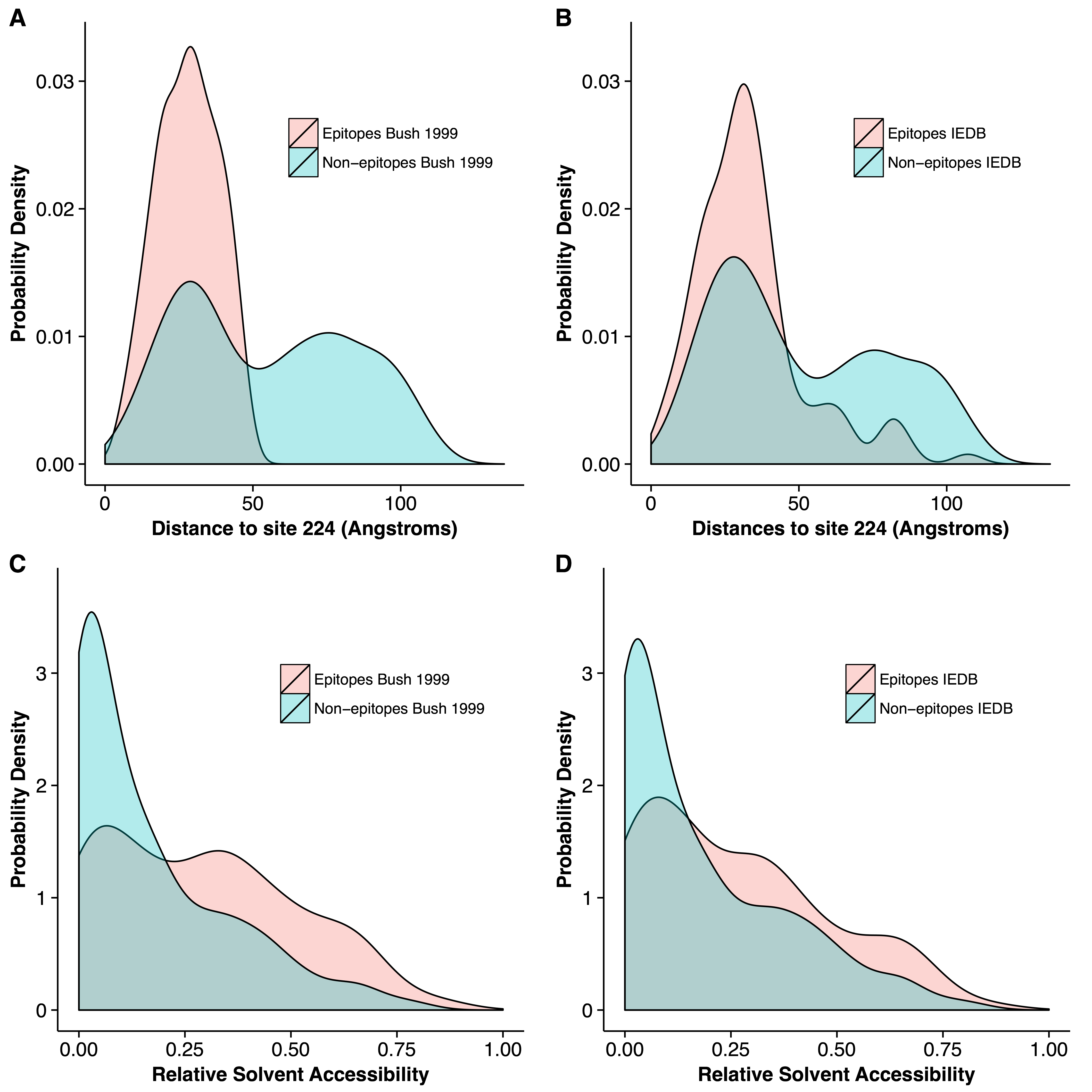

Supplement: S2 Fig — (A) Distribution of distances to residue 224, for historical epitope and non-epitope sites. (B) Distribution of distances to residue 224, for IEDB non-linear epitope and non-epitope sites. (C) Distribution of relative solvent accessibilities, for historical epitope and non-epitope sites. (D) Distribution of relative solvent accessibilities, for IEDB non-linear epitope and non-epitope sites. Under both historical and IEDB epitope definitions, epitope sites are closer to the sialic acid-binding region and have higher RSA than non-epitope sites. (TIFF) [file ppat.1004940.s004.tiff]
